# Poorly Differentiated Ovarian Sertoli-Leydig Cell Tumor in a 16-Year-Old Single Woman: A Case Report and Literature Review

**DOI:** 10.1155/2013/858501

**Published:** 2013-06-25

**Authors:** Ahmed Abu-Zaid, Ayman Azzam, Lama Abdulhamid Alghuneim, Mona Tarek Metawee, Tarek Amin, Turki Omar Al-Hussain

**Affiliations:** ^1^College of Medicine, Alfaisal University, P.O. Box 50927, Riyadh 11533, Saudi Arabia; ^2^Department of General Surgery, Faculty of Medicine, Alexandria University, Alexandria 21526, Egypt; ^3^Department of Surgical Oncology, King Faisal Specialist Hospital and Research Center (KFSH&RC), P.O. Box 3354, Riyadh 11211, Saudi Arabia; ^4^Department of Pathology and Laboratory Medicine, King Faisal Specialist Hospital and Research Center (KFSH&RC), P.O. Box 3354, Riyadh 11211, Saudi Arabia

## Abstract

Sertoli-Leydig cell tumor (SLCT) of ovary is an exceedingly unusual neoplasm that belongs to a group of sex cord-stromal tumors of ovary and accounts for less than 0.5% of all primary ovarian neoplasms. Very few case reports have been documented in the literature so far. Herein, we report a case of primary poorly differentiated ovarian Sertoli-Leydig cell tumor (SLCT) involving the left ovary in a 16-year-old single woman who presented with a 3-month history of a pelviabdominal mass, acne, hirsutism, and menstrual irregularities. In addition, a literature review on ovarian SLCTs is provided.

## 1. Introduction

Sertoli-Leydig cell tumor (SLCT) of ovary is an exceedingly unusual neoplasm that belongs to a group of sex cord-stromal tumor of ovary and accounts for less than 0.5% of all primary ovarian neoplasms [1]. The vast majority of SLCTs are largely diagnosed during reproductive age group (second and third decades of life) [1–4], frequently unilateral, mostly confined to ovary and nearly 90% classified as stage I at the time of clinical diagnosis [5]. Clinical signs and symptoms can be related to either hormonal production (mostly androgen and rarely estrogen) [6] or presence of mass-occupying lesion (mostly pelviabdominal mass and/or pain) [1–3]. Elevated serum levels of testosterone and androstenedione can be identified in approximately 80% of patients with ovarian SLCTs and virilizing manifestations [7, 8]. SLCTs are by far the most common virlizing tumors of ovary [1].

Ultrasound (particularly transvaginal sonography) remains the best imaging modality of preference for initial assessment of adnexal masses [6, 9]. Microscopically, SLCTs are classically made up of uncontrolled proliferation of varying degrees of differentiation of tubules lined by Sertoli cells and intervening nests of Leydig cells [10]. Immunohistochemically, almost all SLCTs, including moderately and poorly differentiated variants, stain positive for inhibin and calretinin, and negative for epithelial membrane antigen (EMA) [11]. A collective profile of hematoxylin and eosin (H&E) stains in addition to immunohistochemical studies are expected to yield the most accurate definitive diagnosis of ovarian SLCTs [4].

Prognosis of ovarian SLCTs is significantly correlated with degree of tumor grading and staging [1]. Management of ovarian SLCTs remains challenging owing to lack of standardized management protocol guidelines [12]. Surgical resection represents the mainstay of management of ovarian SLCTs [4]. The role of postoperative chemotherapy remains questionable and is only indicated in patients with poor prognostic factors [1–3]. 

Herein, we report a case of primary poorly differentiated ovarian Sertoli-Leydig cell tumor (SLCT) involving the left ovary in a 16-year-old single woman who presented with a 3-month history of a pelvi-abdominal mass, acne, hirsutism, and menstrual irregularities. In addition, a literature review on ovarian SLCTs is provided.

## 2. Case Report

A 16-year-old single woman presented to King Faisal Specialist Hospital and Research Center (KFSH&RC) with a 3-month history of a pelvi-abdominal mass, acne, hirsutism, and menstrual irregularities. Past medical history and surgical history were unremarkable. Systemic review was remarkable for decreased appetite, shortness of breath, and constipation. Physical examination revealed acne, hirsutism, abdominal swelling, and a diffuse, tender, and palpable mass extending from lower pelvis/abdomen to umbilicus. 

Laboratory tests showed increased total serum levels of testosterone (T) of 7.8 ng/mL (normal range: 0.2–0.7 ng/mL), dehydroepiandrosterone (DHEA) of 7.2 ng/mL (normal range: 0.8–3.2 ng/mL), and CA-125 of 272 U/mL (normal range <35 U/mL). All other laboratory tests including complete blood count (CBC), renal, bone, hepatic and coagulation profiles, alkaline phosphatase, carcino-embryonic antigen (CEA), alfa-feto protein (AFP), CA 15-3, CA 19-9, estradiol (17*β*-estradiol), luteinizing hormone (LH), follicle-stimulating hormone (FSH), sex hormone-binding globulin (SHBG), adrenocorticotropic hormone (ACTH), prolactin, and cortisol levels were within normal ranges. 

**Figure 1:**
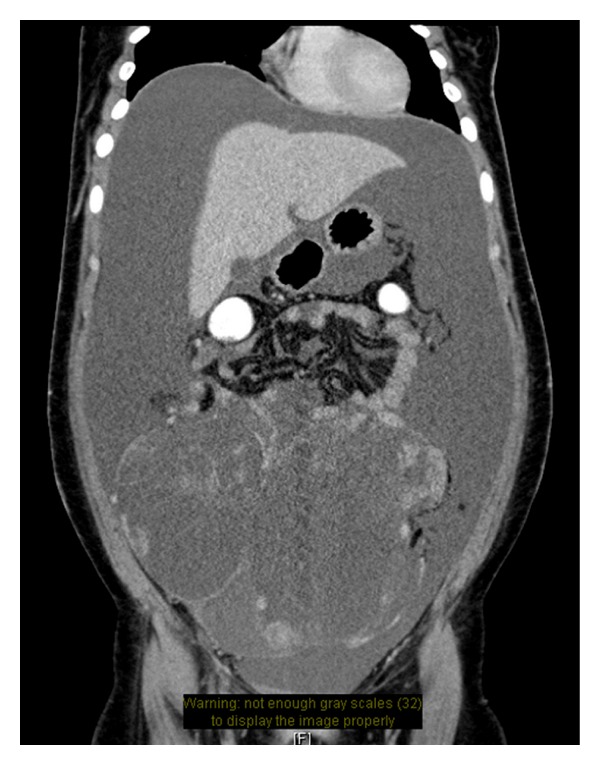
A chest, abdominal, and pelvic contrast-enhanced computed tomography (CT) scan at the portal venous phase with rectal contrast showed a huge, intraperitoneal, complex, cystic, and multilocular lesion, extending from pelvis up to midabdomen just above umbilicus, collectively measuring 13.5 × 23.3 × 21.5 cm. The source of lesion was more likely to be left ovary which was probably ruptured and leaked into the above-mentioned cystic lesion. In addition, large ascites was identified. No significant lymphadenopathies in abdomen and pelvis were noticed.

A chest radiograph (X-ray) showed no evidence of pulmonary nodules. Ultrasound (US) showed intraperitoneal ascites and extremely huge well-vascularized mass with cystic and solid components, mostly arising from left ovary. A chest, abdominal, and pelvic contrast-enhanced computed tomography (CT) scan at the portal venous phase with rectal contrast showed a huge, intraperitoneal, complex, cystic, and multilocular lesion, extending from pelvis up to mid-abdomen just above umbilicus, collectively measuring 13.5 × 23.3 × 21.5 cm. The source of lesion was more likely to be left ovary which was probably ruptured and leaked into the above-mentioned cystic lesion. In addition, large ascites was identified. No significant lymphadenopathies in abdomen and pelvis were noticed (Figure 1). In view of a possible neoplastic process involving the left ovary with androgen-excess manifestations (i.e., virilizing ovarian neoplasm), patient was subjected to laparotomy. 

During laparotomy, frozen section biopsy from the left ovarian mass was consistent with poorly differentiated Sertoli-Leydig cell tumor. Subsequently, the patient underwent left unilateral salpingoophorectomy, omentectomy, and appendectomy. Resected specimens were sent for histopathological evaluation. Macroscopic and microscopic examination of omentum, appendix, and left fallopian tube revealed no significant pathology and were negative for neoplasm.

**Figure 2:**
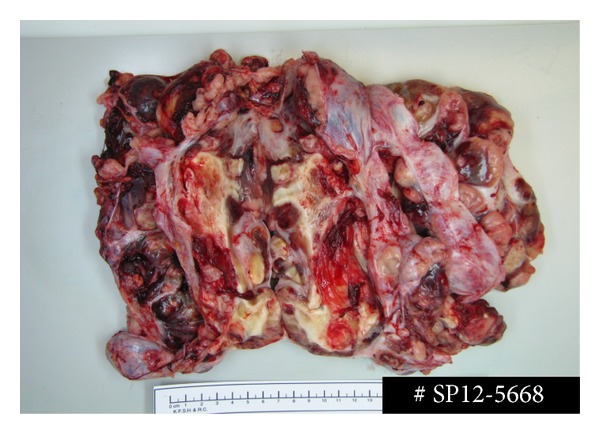
Macroscopic examination of the left ovarian mass. The mass weighed 1945 g and measured 24 × 21 × 7 cm. The mass was well circumscribed and had whitish nodular outer surface. A break of ovarian capsule was noted for a distance measuring 5.5 cm. Cut-surface showed multiple nodules separated by fibrous septa. Some of nodules were necrotic and others had tan-yellow nodular appearance.

Macroscopically, the ovarian mass weighed 1945 g and measured 24 × 21 × 7 cm. The mass was well circumscribed and had whitish nodular outer surface. A break of ovarian capsule was noted for a distance measuring 5.5 cm. Cut-surface showed multiple nodules separated by fibrous septa. Some of the nodules were necrotic and others had tan-yellow nodular appearance (Figure 2).

**Figure 3:**
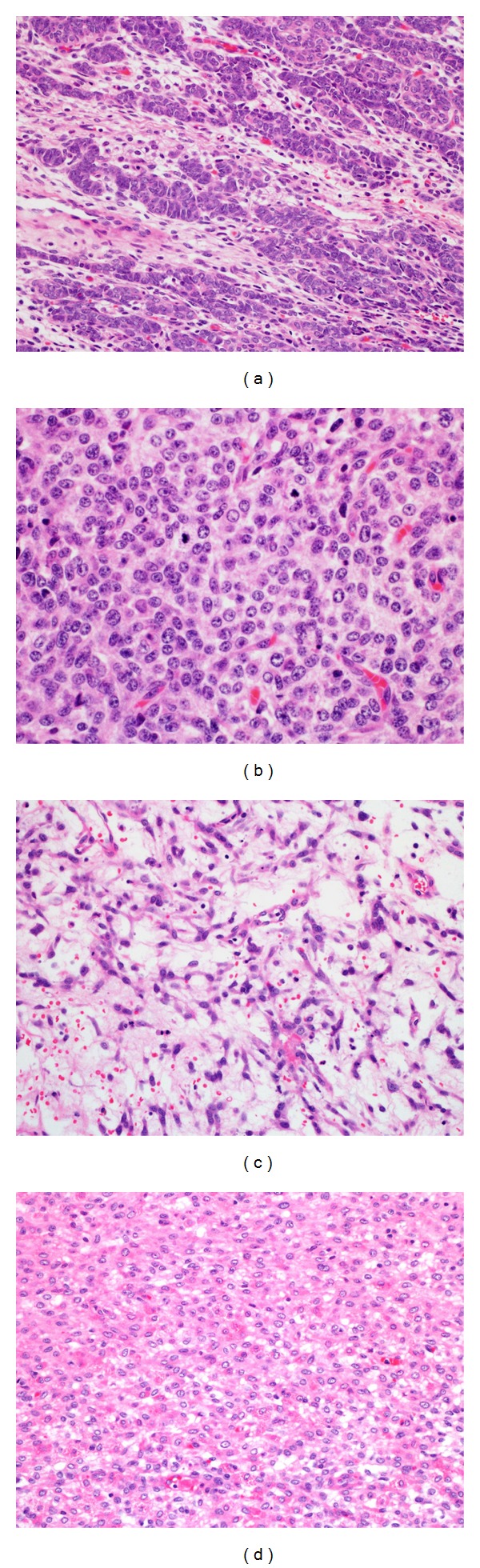
Microscopic examination of the left ovarian mass. (a) The ovarian mass was composed of immature, poorly differentiated, and spindle-shaped Sertoli cells forming cords and ill-defined tubules (H&E stain, magnification power: 20x). (b) The neoplastic Sertoli cells exhibited nuclear atypia and high mitotic indices (H&E stain, magnification power: 40x). (c) Focal myxoid area containing spindle-shaped cells was identified (H&E stain, magnification power: 20x). (d) Infrequent Leydig cells with eosinophilic cytoplasm were identified focally (H&E stain, magnification power: 20x).

Microscopically, the ovarian tumor mass was composed of immature, poorly differentiated, spindle-shaped Sertoli cells forming cords, and ill-defined tubules (Figure 3(a)). The neoplastic Sertoli cells exhibited nuclear atypia and high mitotic index (Figure 3(b)). Focal myxoid area containing spindle-shaped cells was identified (Figure 3(c)). Infrequent Leydig cells with eosinophilic cytoplasm were identified focally (Figure 3(d)). 

**Figure 4:**
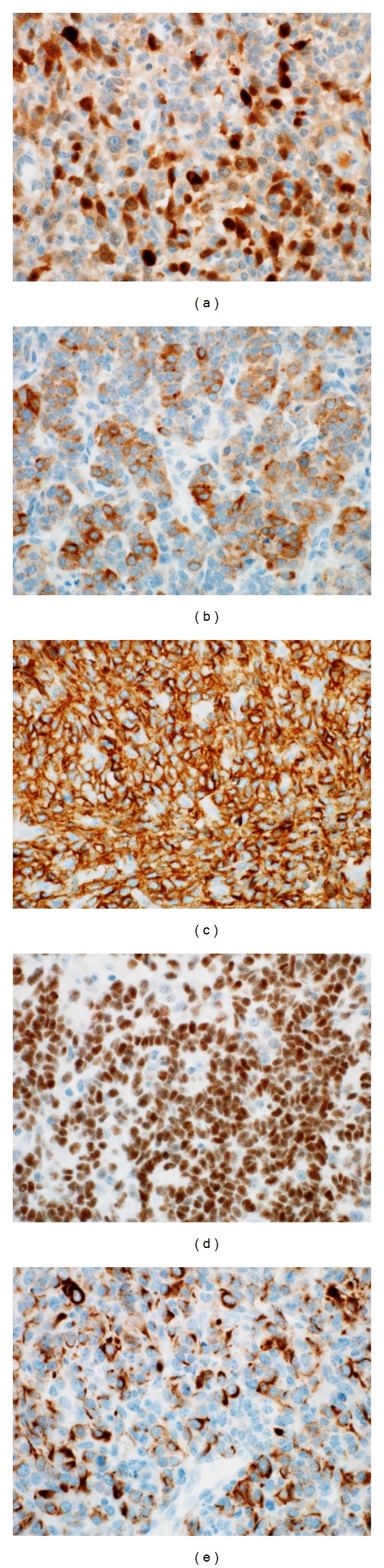
Immunohistochemical examination of the left ovarian mass. (a) The neoplastic cells stained positive for calretinin (magnification power: 40x). (b) The neoplastic cells stained positive for inhibin (magnification power: 40x). (c) The neoplastic cells stained positive for CD56 (magnification power: 40x). (d) The neoplastic cells stained positive for WT-1 (magnification power: 40x). (e) The neoplastic cells stained positive for CK 8/18 (magnification power: 40x).

Immunohistochemically, the neoplastic cells stained positive for calretinin, inhibin, CD56, WT-1, and CK 8/18 (Figures 4(a)–4(e). Conversely, the neoplastic cells stained negative for EMA, PLAP, chromogranin A, and synaptophysin. Based on the clinical, histopathological and immunohistochemical findings, a diagnosis of poorly differentiated ovarian Sertoli-Leydig cell tumor (SLCT) was established.

In consideration of poorly differentiated ovarian SLCT, the patient was considered for adjuvant chemotherapy regimen of bleomycin, etoposide, and cisplatin (BEP). A postoperative 3-month followup failed to show any evidence of recurrence.

## 3. Discussion

Sertoli-Leydig cell tumor (SLCT) of ovary is an exceedingly unusual neoplasm that belongs to a group of sex cord-stromal tumors of ovary and accounts for less than 0.5% of all primary ovarian neoplasms [1]. It is characterized by uncontrolled proliferation of naturally occurring testicular structures (Sertoli and Leydig cells) of varying degrees of differentiation in ovary. The neoplastic Sertoli and Leydig cells exhibit varying degrees of differentiation (grading) which include well differentiated, moderately differentiated, poorly differentiated, and with heterologous elements [13]. 

SLCTs can affect any age group ranging from 2 to 75 years of age. However, 75% of SLCTs take place during second and third decades of life. The average age at the time of clinical diagnosis is roughly 25 years of age. Around less than 10% of SLCTs take place prior to menarche or following menopause [1–3]. Degree of tumor differentiation (grading) seems to be age linked. Patients exhibiting poorly differentiated SLCTs appear to be on average 10 years younger than patients exhibiting well-differentiated SLCTs [1, 3]. The vast majority of SLCTs are frequently unilateral, mostly confined to ovary, and nearly 90% classified as stage I at time of clinical diagnosis [5]. At the time of clinical diagnosis, occurrence of extra ovarian spread of SLCTs is extremely uncommon accounting for roughly 2-3% [1–3]. Moreover, bilateral ovarian SLCTs are exceptionally rare accounting for approximately 1.5–2.0% of all SLCT cases [5].

Clinical signs and symptoms of SLCT can be related to either hormonal production [6] or presence of mass-occupying lesion [1–3]. While SLCTs can be functionally inactive, abnormal hormonal production (mostly androgen or rarely estrogen excess) can be identified in more than half of patients [6]. Clinical expression of virilization is recognized in more than one-third (33–38%) of patients [1, 2, 4]. Androgen-excess manifestations with varying degrees include virilism, hirsutism, hyperseborrhea, acne, receding hairline, alopecia, hoarseness of voice, loss of subcutaneous tissue deposits, breast atrophy, clitoromegaly, oligomenorrhea and amenorrhea. Conversely, although rare, estrogen-excess manifestations include: precocious puberty, abnormal uterine bleeding, abnormal vaginal bleeding, menstrual irregularities, generalized edema, weight gain, breast hypertrophy, endometrial hyperplasia, endometrial polyps and endometrial carcinoma.

Elevated serum levels of testosterone and androstenedione can be often identified in approximately 80% of patients with ovarian SLCTs and virlizing manifestations [7, 8]. Testosterone serum levels greater than 200 ng/dL (7 nmol/L) are generally associated with an androgen-secreting neoplasm from ovaries, adrenals, or elsewhere [14]. Urinary 17-ketosteroid levels are often normal or a bit elevated in patients with SLCTs as opposed to patients with virilizing adrenal tumors who often express extremely elevated levels of urinary 17-ketosteroid levels [1–3]. 

Nearly half of SLCT patients experience symptoms related to growing space-occupying lesions of ovary [1–3]. These symptoms frequently manifest as abdominal/pelvic mass or pain [1–3]. Mass is often adnexal, unilateral, and mobile [1–3]. Mass can be detected by self-examination or clinical (abdominal, vaginal, or rectovaginal) examination [6]. Pain is typically chronic and dull in nature, and occurs secondarily to capsular expansion and possible subsequent compression of nearby visceral structures [6]. Acute abdominal pain requiring prompt emergency intervention happens in roughly less than one-fifth (15%) of SLCT cases and can be attributable to ovarian torsion, capsular rupture, or bleeding [6].

Imaging studies can be utilized in the diagnosis of ovarian SLCTs. Sonography (ultrasound) remains the best imaging modality of preference for initial assessment of adnexal masses, due to its high sensitivity, suitability, and cost-effectiveness [6, 9]. Transvaginal sonography, as opposed to abdominal sonography, appears to yield better morphologic features of adnexal masses [15]. SLCTs typically exhibit solid sonographic appearance [6, 9] and mostly unilateral tumors [1–3, 5]; bilateral tumors are exceptionally uncommon [5]. Components of SLCTs can be purely solid, purely cystic, or mixed [1]. Mixed (solid and cystic) components are most commonly encountered in roughly 60% of all ovarian SLCTs [1]; pure cystic ovarian SLCTs are extremely unusual [1, 6]. Average SLCT diameter is 13.5 cm and can reach as huge as 50 cm in poorly differentiated histological variants [1]. In the settings of clinical and laboratory proofs of androgen or estrogen excess, a normal sonographic study does not rule out diagnosis of ovarian SLCT, as the size of tumor can sometimes be undetectable by sonography [6]. Color Doppler sonography offers further categorization and evaluation of neoplastic masses. Moderate-to-rich ovarian vascular masses with low-resistance indices highly suggest malignant rather than benign lesions [16]. Other imaging modalities such as computed tomography (CT), magnetic resonance imaging (MRI), and positron imaging tomography (PET) scans can be used for better characterization of ovarian SLCTs, detection of extraovarian disease/metastasis and identification of other possible primary neoplasms (e.g., a functional androgen-producing adrenal gland tumor). 

Macroscopically [10], SLCTs are frequently unilateral, well-encapsulated, solid, firm, lobulated, and yellow-gray masses of roughly 7 cm in diameter on average. Cut-section surface exhibits varying degrees of greasy/fleshy consistency, straw-colored fluid, necrosis, hemorrhage, and cystic spaces separated by fibrous septae. 

Microscopically [10], SLCTs are classically made up of uncontrolled proliferation of varying degrees of differentiation of tubules lined by Sertoli cells and intervening nests of Leydig cells. Well-and moderately differentiated SLCTs are the most frequently encountered histological variants. Leydig cells are typically found in clusters in interstitial stroma and typically exhibit polygonal cells with well-defined margins, centric nuclei, prominent nucleoli, and eosinophilic cytoplasm. Sertoli cells typically form tubular structures lined by single or multiple layers of cuboidal-columnar cells with well-bounded margins, oval dark (basal) nuclei, inconspicuous nucleoli and eosinophilic or vacuolated cytoplasm. Mitotic figures are extremely rare. Poorly differentiated SLCTs, as in our case report, represent a considerable diagnostic challenge owing to the huge range of microscopic/histopathological diversity. The classical arrangement of tubules lined by Sertoli cells and intervening nests of Leydig cells is greatly minimal, very occasional and most often difficult to identify. The sex-cord neoplastic cells exhibit immature (poor) differentiation with high nuclear atypia, increased nuclear-to-cytoplasmic ratio, coarse chromatin and extremely abundant mitotic figures. For histopathologists with low index of suspicion of ovarian SLCTs, all these microscopic features can be misleading and easily mistaken for a diagnosis of undifferentiated sarcoma.

Immunohistochemically, almost all SLCTs, including moderately and poorly differentiated variants, stain positive for inhibin and calretinin, and negative for epithelial membrane antigen (EMA) [11]. In addition, it has been shown that SLCTs stain positive for WT-1 [17] and CD56 [18]. A collective profile of hematoxylin and eosin (H&E) stains in addition to immunohistochemical studies are expected to yield the most accurate definitive diagnosis of SLCTs [4]. 

Management of ovarian SLCTs remains challenging owing to lack of standardized management protocol guidelines [12]. Surgical resection represents the mainstay of management of ovarian SLCTs [4]. Fortunately, the vast majority of SLCTs are largely diagnosed during reproductive age [1–4], frequently unilateral, mostly confined to ovary and nearly 90% classified as stage I at time of clinical diagnosis [5]. Therefore, fertility-sparing surgery (unilateral salpingooophorectomy) can be considered in all patients with well-differentiated ovarian SLCTs. Patients desiring fertility and exhibiting moderately or poorly differentiated ovarian SLCTs can be considered for unilateral salpingo-oophorectomy plus standard staging surgery (omentectomy, appendectomy, and pelvic lymphadenectomy) [12, 19]. The need for pelvic lymphadenectomy is still debatable. However, the widely accepted conclusion is that pelvic lymph node metastasis is extremely rare in ovarian SLCTs and therefore, pelvic lymphadenectomy may be excluded during the staging surgery [20]. Patients who are elderly, not desiring fertility or with progressively advanced SLCTs should be considered for total hysterectomy, bilateral salpingooophorectomy in addition to complete standard staging surgery [18].

Owing to rarity of ovarian SLCTs, limited number of documented case reports/series and lack of randomized clinical trials, effectiveness of post-operative chemotherapy remains questionable and requires further evaluation [4, 12, 19, 21]. Generally, postoperative chemotherapy is considered for patients with poor prognostic factors such as: advanced disease staging, moderate-to-poor tumor grading, high mitotic profile, existence of heterologous elements and tumor rupture [1–3]. The first-line and most frequently used chemotherapeutical regimen is bleomycin, etoposide, and cisplatin (BEP) [22, 23]. Other regimens also exist such as (1) cisplatin, Adriamycin, and cyclophosphamide (CAP), and (2) cisplatin, vinblastine, and bleomycin (PVB) [24].

Prognosis of ovarian SLCTs is significantly correlated with degree of tumor differentiation (grading) and tumor extent (staging) [1]. Well-differentiated (grade 1) SLCTs are associated with zero malignant potential, whereas moderately (grade 2) and poorly (grade 3) differentiated SLCTs are associated with 11% and 59% malignant potential respectively [1]. The overall 5-year survival rate for well-differentiated (grade 1) SLCTs is 100%, whereas for moderately (grade 2) and poorly (grade 3) differentiated SLCTs is collectively 80% [21]. With respect to tumor staging, the overall 5-year survival rate for stage I is 95% [2] whereas for stages III and IV is nearly zero percent [1]. Long-term followup is highly advised in all patients.

## References

[1] Young RH, Scully RE (1985). Ovarian Sertoli-Leydig cell tumors. A clinicopathological analysis of 207 cases. *American Journal of Surgical Pathology*.

[2] Zaloudek C, Norris HJ (1984). Sertoli-Leydig tumors of the ovary. A clinicopathologic study of 64 intermediate and poorly differentiated neoplasms. *American Journal of Surgical Pathology*.

[3] Roth LM, Anderson MC, Govan ADT (1981). Sertoli-Leydig cell tumors: a clinicopathologic study of 34 cases. *Cancer*.

[4] Weng CS, Chen MY, Wang TY (2013). Sertoli-Leydig cell tumors of the ovary: a Taiwanese gynecologic oncology group study. *Taiwanese Journal of Obstetrics & Gynecology*.

[5] Young RH, Scully RE, Kurman RJ (2002). Sex cord-stromal, steroid cell, and other ovarian tumors. *Blaustein's Pathology of Female Genital Tract*.

[6] Zanotti KM (2002). The clinical manifestations and diagnosis of Sertoli-Leydig cell tumors of the ovary. *CME Journal of Gynecologic Oncology*.

[7] Osborn RH, Yannone ME (1971). Plasma androgens in the normal and androgenic female: a review. *Obstetrical & Gynecological Survey*.

[8] Prunty FT (1967). Hirsutism, virilism and apparent virilism and their gonadal relationship. II. *Journal of Endocrinology*.

[9] de Oliveira Franzin CM, Kraft ML, Faundes D, Zeferino LC, Alvarenga M, Marussi EF (2006). Detection of ovarian Sertoli-Leydig cell tumors exclusively by color Doppler sonography. *Journal of Ultrasound in Medicine*.

[10] Nouriani M, Felix JC, Dubeau L (2002). Histogenesis and histopathological characteristics of Sertoli-Leydig cell tumors. *CME Journal of Gynecologic Oncology*.

[11] McCluggage WG, Young RH (2005). Immunohistochemistry as a diagnostic aid in the evaluation of ovarian tumors. *Seminars in Diagnostic Pathology*.

[12] Bhat RA, Lim YK, Chia YN, Yam KL (2013). Sertoli-Leydig cell tumor of the ovary: analysis of a single institution database. *Journal of Obstetrics and Gynaecology Research*.

[13] Chen VW, Ruiz B, Killeen JL, Coté TR, Wu XC, Correa CN (2003). Pathology and classification of ovarian tumors. *Cancer*.

[14] Meldrum DR, Abraham GE (1979). Peripheral and ovarian venous concentrations of various steroid hormones in virilizing ovarian tumors. *Obstetrics and Gynecology*.

[15] Mendelson EB, Bohm-Velez M, Joseph N, Neiman HL (1988). Gynecologic imaging: comparison of transabdominal and transvaginal sonography. *Radiology*.

[16] Yanushpolsky EH, Brown DL, Smith BL (1995). Localization of small ovarian Sertoli-Leydig cell tumors by transvaginal sonography with color Doppler. *Ultrasound in Obstetrics & Gynecology*.

[17] Cathro HP, Stoler MH (2005). The utility of calretinin, inhibin, and WT1 immunohistochemical staining in the differential diagnosis of ovarian tumors. *Human Pathology*.

[18] McCluggage WG, McKenna M, McBride HA (2007). CD56 is a sensitive and diagnostically useful immunohistochemical marker of ovarian sex cord-stromal tumors. *International Journal of Gynecological Pathology*.

[19] Gui T, Cao D, Shen K (2012). A clinicopathological analysis of 40 cases of ovarian Sertoli-Leydig cell tumors. *Gynecologic Oncology*.

[20] Brown J, Sood AK, Deavers MT, Milojevic L, Gershenson DM (2009). Patterns of metastasis in sex cord-stromal tumors of the ovary: can routine staging lymphadenectomy be omitted?. *Gynecologic Oncology*.

[21] Sigismondi C, Gadducci A, Lorusso D (2012). Ovarian Sertoli-Leydig cell tumors. A retrospective MITO study. *Gynecologic Oncology*.

[22] Chen FY, Sheu BC, Lin MC, Chow SN, Lin HH (2004). Sertoli-Leydig cell tumor of the ovary. *Journal of the Formosan Medical Association*.

[23] Sachdeva P, Arora R, Dubey C, Sukhija A, Daga M, Singh DK (2008). Sertoli-Leydig cell tumor: a rare ovarian neoplasm. Case report and review of literature. *Gynecological Endocrinology*.

[24] Roth BJ, Greist A, Kubilis PS, Williams SD, Einhorn LH (1988). Cisplatin-based combination chemotherapy for disseminated germ cell tumors: long-term follow-up. *Journal of Clinical Oncology*.

